# Exploiting immunostimulatory mechanisms of immunogenic cell death to develop membrane-encapsulated nanoparticles as a potent tumor vaccine

**DOI:** 10.1186/s12951-023-02031-w

**Published:** 2023-09-08

**Authors:** Qingwen Liu, Yongmao Hu, Peng Zheng, Ying Yang, Yuting Fu, Ying Yang, Biao Duan, Mengzhen Wang, Duo Li, Weiran Li, Jinrong He, Xiao Zheng, Qiong Long, Yanbing Ma

**Affiliations:** 1 Laboratory of Molecular Immunology, Institute of Medical Biology, Chinese Academy of Medical Sciences & Peking Union Medical College, Kunming, 650118 China; 2 Institute of Medical Biology, Kunming Medical University, Kunming, 650500 China; 3 School of Life Sciences, Yunnan University, Kunming, 650091 China

**Keywords:** Tumor vaccine, Immunogenic cell death, Cell membrane, Biomimetic nanoparticles

## Abstract

Vaccine is one of the most promising strategies for cancer immunotherapy; however, there are no therapeutic cancer vaccine achieving significant clinical efficacy till now. The main limiting factors include the immune suppression and escape mechanisms developed by tumor and not enough capacity of vaccines to induce a vigorous anti-tumor immunity. This study aimed to develop a strategy of membrane-based biomimetic nanovaccine and investigate the immunological outcomes of utilizing the unique immunostimulatory mechanisms derived of immunogenic cell death (ICD) and of fulfilling a simultaneous nanoscale delivery of a highlighted tumor antigen and broad membrane-associated tumor antigens in the vaccine design. TC-1 tumor cells were treated in vitro with a mixture of mitoxantrone and curcumin for ICD induction, and then chitosan (CS)-coated polylactic co-glycolic acid (PLGA) nanoparticles loaded with HPV16 E7_44-62_ peptides were decorated with the prepared ICD tumor cell membrane (IM); further, the IM-decorated nanoparticles along with adenosine triphosphate (ATP) were embedded with sodium alginate (ALG) hydrogel, And then, the immunological features and therapeutic potency were evaluated in vitro and in vivo. The nanovaccine significantly stimulated the migration, antigen uptake, and maturation of DCs in vitro, improved antigen lysosome escape, and promoted the retention at injection site and accumulation in LNs of the tumor antigen in vivo*.* In a subcutaneously grafted TC-1 tumor model, the therapeutic immunization of nanovaccine elicited a dramatical antitumor immunity. This study provides a strategy for the development of tumor vaccines.

**Graphical Abstract:**

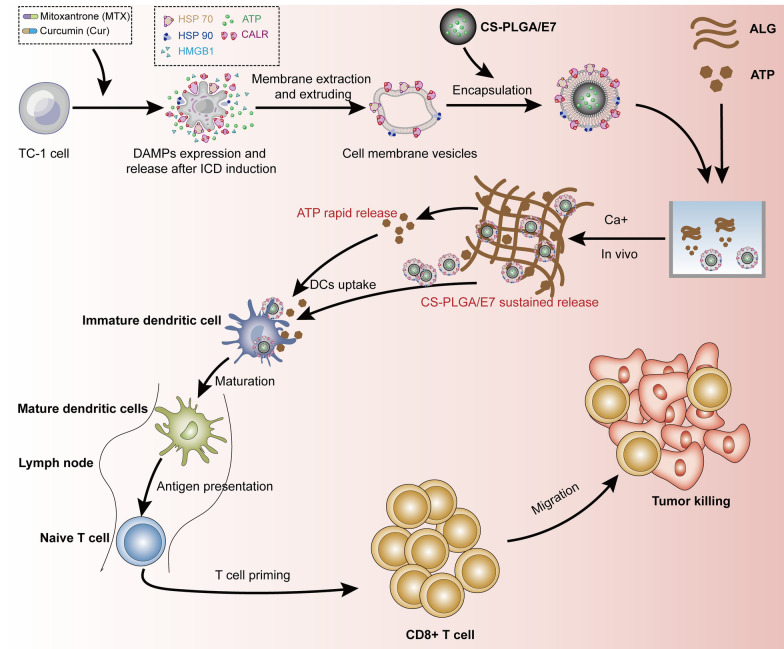

**Supplementary Information:**

The online version contains supplementary material available at 10.1186/s12951-023-02031-w.

## Introduction

Tumor vaccines, which is designed to recognize and eradicate the tumor cells by activating the patient’s own immune system, have a good clinical prospect [1, 2]; however, there are still important obstacles to therapeutic successes at present. The tolerogenicity, heterogenicity, and immune evasion of tumor antigen and tumor immune suppression mechanisms make the vaccine design challenging.

One of the conventional and attractive ideas is to develop a vaccine based on tumor cells, which can provide a broad spectrum of tumor antigens; however, the existence of a mass of irrelevant and even harmful components may intervene the immune responses [3, 4]. Instead, the membrane derived from tumor cells was proposed as a vaccine candidate, which does not contain genetic materials and may enrich tumor associated antigens (TAA) [5–7]. In recent years, the application of nanobiotechnology in vaccine delivery develops rapidly, and the strategies of using tumor cell membrane to decorate nanoparticles (NPs) have attracted increasing attention [8–10]. Nanoparticles act as a physical supporter for the cell membrane, providing a nano-sized structure, protecting loaded substances from enzymatic degradation, and forming antigen and adjuvant deposit for a controlled release. Polylactic co-glycolic acid (PLGA) is a FDA approved biodegradable copolymer for clinical applications and its nanoparticle has great potentiality to be used as a vaccine carrier [11, 12]. It was reported that PLGA NPs loaded with adjuvants such as imiquimod and CpG1826 and further coated with tumor cell membranes achieved a nanoscale co-deliver of tumor cell membrane antigens and adjuvants, and elicited an anti-tumor immune response in a mouse 4T-1 breast carcinoma model or B16-F10 melanoma model respectively [13]. Activation and maturation of dendritic cells (DCs) is a pre-requisite for vaccine induction of an effective cell-mediated anti-tumor immune response [3, 14]. Hu et al. prepared the membranes of B16 melanoma cells modified with the highly immunostimulatory agent polyinosinic: polycytidylic acid (poly-IC), which effectively enhanced DC activation and antigen cross-presentation and induced anti-tumor cellular immunity [15]; Liu et al*.* modified tumor cell membranes with mannose, which promoted the binding and uptake of the NPs by APCs [16]. Gao et al. developed a strategy by preparing peptide CBP-12-expressing tumor cell membrane to encapsulate PLGA NPs and targeting the NPs to DCs through the interaction of CBP-12 peptide and Clec9a on DC surface, and they also co-delivered STING agonists and gained synergistic antitumor effects [17]. In addition, membrane fusion strategy was also developed for functionally modifying the membrane-encapsulated NPs. For example, the membrane derived from the primary tumor tissue was fused with *E. coli* cytoplasm membrane to encapsulate NPs or with outer membrane vesicles to form nano-vesicles, which produced significant anti-tumor effects [18, 19] Although some attempts of modifying tumor cell membrane and loading immunostimulatory agents to enhance the vaccine efficacy have been performed, considering the possible challenges for the synthesizing efficiency, the stability, the modification procedure and the actually immunological properties and safety of the strategic cell membrane surface ligands, the searching for more modification strategies of tumor cell membrane ligands keeps quite attractive.

Immunogenic cell death (ICD) is a form of programmed cell death triggered by chemotherapy drugs, lytic viruses, physical chemotherapy, photodynamic therapy and radiation therapy, etc. The cells occurring ICD increase the expression and the release or translocation of damage-associated molecular patterns (DAMPs) and proinflammatory cytokines, which produce a strong stimulus to immune system [20–24]. The DAMPs include high mobility group protein B1 (HMGB1) and “find me” signals adenosine triphosphate (ATP), which facilitates the recruitment of DCs and other innate immune cells and “eat me” signals Calreticulin protein (CARL), the heat shock proteins HSP70 and HSP90, which deliver phagocytic signals to professional antigen-presenting cells (APCs), improving their ability of uptake and presentation of antigens [25–29].

In this paper, we try to explore a new membrane-based biomimetic nanovaccine, employing a nanoparticle/hydrogel system, exploiting the unique immunostimulatory mechanisms of tumor cell ICD, and fulfilling a co-delivery of a key tumor-specific antigen encapsulated in NPs and a spectrum of tumor antigens provided by ICD cell membrane coating on NPs surface.

## Materials and methods

### Reagents

**Scheme 1:**
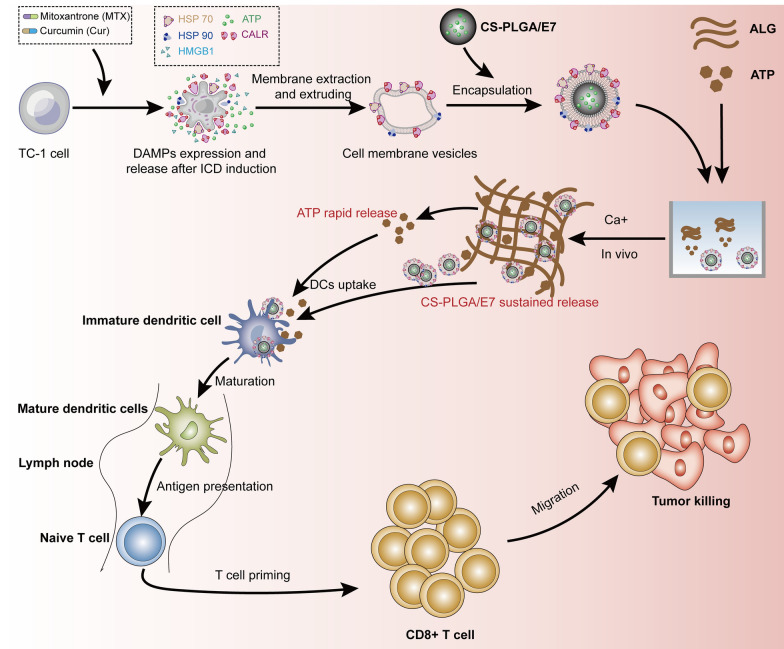
Schematic illustration for the preparation of IM-CS-NPs/E7/ATP@ALG nanovaccine and its effects inducing antitumor immunity

Lactic acid-ethanolic acid (PLGA) copolymer 50/50 was obtained from Jinan Daigang Bioengineering Co; chitosan and PVA were purchased from Sigma; the Flow cytometry antibodies were provided by Biolegend, USA; LysoTracker Red were provided by Solarbio (L8010). DAPI Stain Solution (ab104139), Na^+^/K^+^-ATPase antibody (ab76020), HMGB1 antibody (ab79823), Donkey Anti-Rabbit IgG H&L antibody (ab150075) and Goat Anti-Rabbit IgG H&L antibody (ab150077) were purchased from Abcam; the antibodies for Calreticulin (27298–1-AP), HSP70 (10995–1-AP), HSP90 (13171–1-AP), HRP Goat Anti-Rabbit IgG antibody (SA00001-2) and ELISA kits (IL-1β, IL6, TNF-α and IFN-γ) were purchased from Proteintech (Scheme 1).

### Mouse and cell lines

Female C57BL/6 mice (6–8 weeks) were provided by the Central Animal Care Services of the Institute of Medical Biology, Chinese Academy of Medical Sciences (CAMS) and PUMC (Kunming, China). All the mice had access to food and water ad libitum. All mice were maintained in a specific pathogen-free environment. The animal procedures were performed with ethical compliance and approval by the Animal Care and Welfare Ethics Committee, Institute of Medical Biology, CAMS (ethics number: DWSP202004026).

TC-1 cells (Tumor Cell Bank, CAMS) are lung epithelial cells of C57BL/C (H-2b) mice cotransfected with HPV16 E6 and E7 and the ras gene, which has been widely used to establish a mouse model of HPV-related tumors for assessing the efficacy of therapeutic vaccines [30]. The cells were cultured in RPMI1640 complete medium containing 100 μg/mL streptomycin, 1% 100 U/mL penicillin and 10% fetal bovine serum, at 37 °C, in a 5% CO_2_ thermostatic cell incubator.

### Peptides

Seven peptides were synthesized by GL Biochem Co, Ltd. (Shanghai, China). HPV16 E7_49–57_(^49^RAHYNIVTF^57^) and a mixture of E7 peptides including HPV16 E7_11-20_ (^11^YMLDLQPETT^20^), HPV16 E6_29-38_ (^29^TIHDIILECV^38^), HPV16 E6_49-58_ (^49^VYDFAFRDLC^58^), and HPV16 E7_67-75_ (^67^LCVQSTHVD^75^) were used for stimulating cultured lymphocytes in vitro*.* HPV16 E7_44–62_ (^44^QAEPDRAHYNIVTFCCKCD^62^), which contains an identified T helper cell (Th) epitope and an important CTL epitope recognized by C57BL/6 mice, was used as a representative tumor antigen for constructing PLGA nanoparticle vaccines and assessing their efficacy in HPV-related TC-1tumor model, and Fluorescein isothiocyanate (FITC)-tagged E7_44–62_ was used for fluorescence localization of CS-NPs. All peptides were more than 98% pure.

### Detection of the indicators of immunogenic cell death

The same number of TC-1 tumor cells at a concentration of 1 × 10^6^ cells/mL were inoculated into each well of a 6-well cell culture plate 1 day in advance and then treated with a mixture of 3 μmol/L mitoxantrone (MTX) and 50 μmol/L curcumin (Cur). The equal volume of culture supernatants were collected at designated time points for the measurement of the released ATP and HMGB1. The ATP concentration was detected with ATP kit and HMGB1 was measured by Western blotting. In addition, the expression and membrane translocation of “Eat Me” signals CALR, HSP70 and HSP90 in the cells of immunogenic death were analyzed with immunofluorescence assay, and the images were recorded under a confocal microscope. In addition, the cells were collected for flow cytometry analysis.

### Isolation of cell membranes

The collected cells received freeze-thawing cycles for 3–4 times by putting in liquid nitrogen and back to room temperature repeatedly. The cell suspension was centrifuged at 5000 g for 15 min at 4 °C to remove the nucleus pellet and the supernatant was further centrifuged at 15,000 g for 45 min at 4 °C to precipitate cell membrane debris and then stored at − 80 °C.

### Preparation of chitosan -modified NPs

The E7 peptide-loaded and chitosan-decorated PLGA NPs were prepared by solvent evaporation. Briefly, 6 mL of dichloromethane (DCM) organic solvent was added to 180 mg of PLGA to form the oil phase, E7_44-62_ or FITC-E7_44-62_ (1 mg/mL) was added as the first aqueous phase W1 to the oil phase, and then the mixture was ultrasonicated for 1 min an of interval 5 s at 4 °C to produce a primary emulsion W1/O, followed by being mixed with 24 mL of the second aqueous phase W2 (2% PVA + 0.5% CS) and sonicated for 5 min to form a second emulsion W1/O/W2; after stirring overnight to allow DCM to evaporate completely, the NPs were obtained by centrifugating at 21,000 rpm for 30 min and washing three times with distilled water; Finally, the precipitate was resuspended in phosphate-buffered saline (PBS).

### Preparation of the hydrogel-embedded ICD cell membrane-encapsulated PLGA NPs

For preparing the membrane -decorated NPs, the isolated IM were extruded through a 400 nm porous polycarbonate membrane (LiposoFast-1, Avestin, Canada) to form IM vesicles, and then the vesicles and CS-NPs/E7 were mixed and passed through a 200 nm porous polycarbonate membrane for a total of 15 cycles. Further, ATP and the prepared membrane-encapsulated NPs was mixed in the solution at 10 mg/mL of ALG working concentration.

### Characterization of the membrane-encapsulated biomimetic NPs

The morphology was observed by a transmission electron microscope (model: HITACHI H-7650). The size distribution and surface potential were analyzed by dynamic light scattering (DLS) with Zetasizer NanoZS (Malvern).

The encapsulation rate (EE) of E7_44-62_ by CS-NPs were determined using an indirect quantitative method, by detecting the fluorescence intensity in the supernatant of the second emulsion using BioTek Gen5, and EE was calculated with a formula: EE = 100% × (initial E7_44-62_ amount – unencapsulated free E7_44-62_ amount)/initial E7_44-62_ amount.

For indicating that the membrane has covered successfully onto the NPs, tumor cell membranes obtained from immunogenic cell death were stained with DID dye on a shaker at 25 °C for 1 h, FITC-E7 was encapsulated by PLGA NPs as a tracker, and the DID-IM and CS-NPs/FITC-E7 NPs were used to prepare the labeling membrane-encapsulated NPs. The preparation was observed by CLSM confocal laser scanning microscopy.

To characterize the formation of hydrogel, the different concentrations of calcium ions (0 mg/mL, 5 mg/mL, 10 mg/mL and 15 mg/mL) were mixed with CS-NPs/E7/ATP@ALG in vitro and the transition from solution to hydrogel were observed. Further, to evaluate the kinetics of E7 peptide and ATP release from IM-CS-NPs/E7/ATP@ALG, IM-CS-NPs/E7/ATP and IM-CS-NPs/E7/ATP@ALG were placed in dialysis bags (7 kDa; Biosharp) separately, and the cumulative release concentration of E7 antigen was determined by the Micro-BCA Protein Assay Kit and that of ATP was determined by the ATP Assay Kit.

### Migration of BMDCs

Briefly, bone marrow cells isolated from the femurs and tibias of 7-week-old C57BL/6 mice were incubated in RPMI-1640 medium with 10% FBS and recombinant granulocyte–macrophage colony-stimulating factor (GM-CSF, 20 ng/mL, PeproTech). On the 5th day, the medium was replaced with fresh medium containing GM-CSF, and the BMDCs were harvested in 2 days. Further, 200 μL of 1 × 10^5^ cells were added to the upper chamber of Transwell with a membrane pore size of 8 μm (Corning), and 500 μL of the samples, were added to the lower chamber respectively. After incubation for 8 h, The cells were observed under the microscope.

### Uptake of the NPs by DC2.4 cells

IM was labeled with DID (DID-IM) and CS-NPs were loaded with FITC-E7_44-62_ (CS-NPs/FITC-E7), and then they were mixed and extruded through an extruder to obtain DID-IM-CS-NPs/FITC-E7. The established cell line DC2.4 cells (5 × 10^5^ cells/mL) were spread in 6-well plates 1 day in advance and then incubated with 100 μL of CS-NPs/FITC-E7 and IM-CS-NPs/FITC-E7 NPs for 0.5 h and 4 h respectively. The fluorescence signals were examined with confocal laser scanning microscopy (CLSM).

### Activation and maturation of BMDC cells

BMDCs were inoculated into 96-well plates at 1 × 10^6^ cells/well with a volume of 100 μL, and incubated with the samples, with PBS and LPS (10 μg/mL) as negative and positive controls respectively for 24 h. The cytokine concentrations in the supernatant were measured by enzyme-linked immunosorbent assay (ELISA), and the expression of surface markers in DCs were analyzed by flow cytometry using FITC-CD11c, APC-CD80, APC-CD86, APC-H-2 Kb (MHC I) and APC-I-A/I-E (MHC II) antibodies. Data were collected by a CytoFLEX flow cytometer (Beckman Coulter, USA).

### Lysosomal escape

BMDCs were inoculated into 6-well plates at 2 mL of 1 ◊ 10^6^ cells/well. After incubation for 24 h, cells were incubated with 100 μL samples. and PBS as a control. At designated timepoints, the imaging was observed and recorded with a confocal microscope.

### Biodistribution of the antigen in vivo*.*

C57BL/6 mice are injected subcutaneously *(s c.)* with 100 μL samples. ITC fluorescence signal at the injection site was detected by the In-Fino FX PRO living imaging system (BRUKER, USA) at different time points. In a parallel experiment, mice were euthanized 8 h after the injection, and the major organs including heart, liver, spleen, lungs, kidneys and inguinal lymph nodes were collected for ex vivo fluorescence imaging.

### Tumor model and immunization

A HPV infection-related tumor model, which provides well defined and virus -derived tumor-specific antigens, was established through subcutaneous transplantation of TC-1 cells in C57BL/6, for which 100 μL of the mixture of tumor cells (5 × 10^5^ cells/100 μL) and matrigel were inoculated subcutaneously on the right back of the mice. When the tumor diameter reached the experimental requirement, mice were immunized subcutaneously on the left back with 100 μL PBS or the nanovaccine formulations, containing 4 μg E7 antigen, 30 μg IM and 10 μM ATP, per mouse for three times at 1 week interval. And a regular monitoring of tumor growth was performed using vernier calipers. Before the tumors size reached to the ethical stipulation, the mice were euthanized and the spleen and tumor were collected for weighing and immunological testing.

### ELISPOT assay

Briefly, 5 × 10^5^ cells/100 μL splenic lymphocytes were stimulated with the E7_49-57_ peptide, mixture E7 peptides and ICD membrane components at a concentration of 5 mg/mL. After 48 h of incubation, IFN-γ roducing cells were determined with an IFN-γ ELISPOT assay kit following the manufacturer’s protocol (MABTECH). Visualized spots were enumerated with the ELISPOT Reader System (AID).

### Flow cytometry

The lymphocytes were isolated from spleen using mouse lymphocyte separation medium. The cells were incubated in vitro and stimulated with E7_49-57_ peptide, ICD membrane components, or a mixture of E7 peptides at a protein concentration of 5 mg/mL. After 6 h of incubation, the cells were labeled with FITC-anti-mouse CD3, APC-anti-mouse CD8α, and PE-anti-mouse Gr-1 and APC-anti-mouse CD11b antibodies for staining CD8^+^ cells and MDSCs respectively. The data were recorded by CytoFLEX flow cytometer (Beckman Coulter, USA) and analyzed using FlowJo software.

### Preliminary safety evaluation

The mice were injected for three times with 100 μL IM-CS-NPs/E7/ATP@ALG, with PBS as a control, which is consistent with the protocol used for immunization experiment. The blood was collected and tested for the biochemical parameters including alanine aminotransferase (ALT), aspartate transaminase (AST), urea nitrogen (BUN) and lactate dehydrogenase (LDH) using an automated analyzer (Chemray—800). In addition, the major organs were collected, fixed in formalin, embedded with wax, and sectioned for hematoxylin—eosin staining (H&E).

### Statistical analysis

All values were reported as the mean ± standard error of the mean (SEM). Two-way ANOVA was used to analyze dynamic tumor growth, one-way ANOVA was used when more than two groups were compared, and Student’s t-test was used for comparisons between two-group. All statistical analyses were carried out with GraphPad Prism 8.0 software. Significant p values were denoted by **** *P* < 0.0001, *** *P* < 0.001, ** *P* < 0.01 and * *P* < 0.05; ^##^ *P* < 0.01, ^#^ *P* < 0.05.

## Results

### Tumor cells present immunostimulatory signals after the induction of immunogenic cell death

**Fig. 1:**
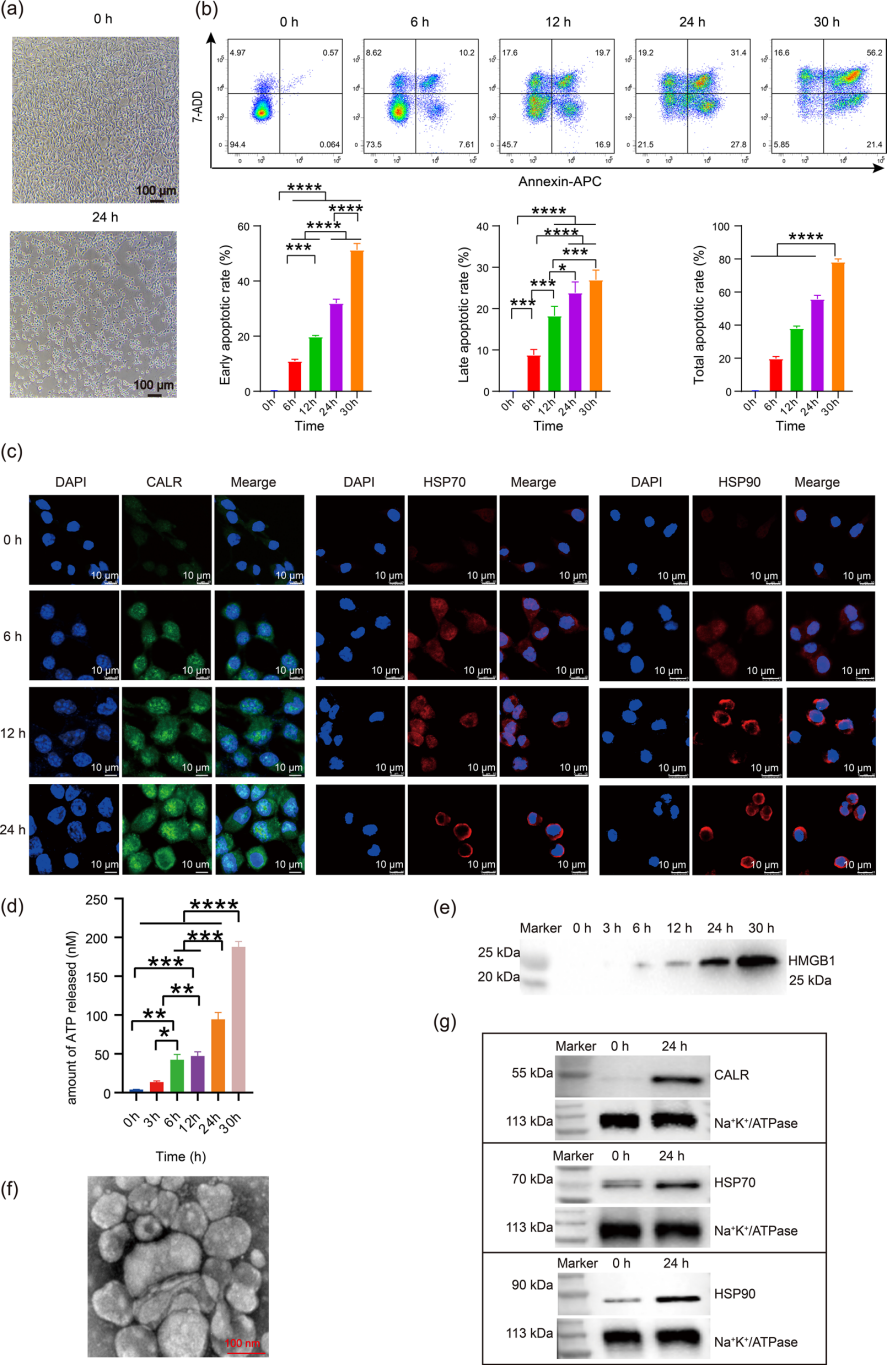
The induction of immunogenic cell death and membrane vesicle preparation of TC-1 tumor cells. **a** Observation of the morphology of TC-1 cells treated with the mixture of MTX and Cur for 24 h. **b** The analysis on cell apoptosis by flow cytometry. upper panel, representative scatter plot of flow cytometry analysis; lower panel, statistical analyses of early apoptosis, late apoptosis and total apoptosis. **c** The expression and membrane translocation of “eat me” signals visualized by immunofluorescence at 6 h, 12 h and 24 h. **d** The release of “find me” signal ATP in the supernatant of treated tumor cells (n = 3). **e** The release of “find me” signal HMGB1 in the supernatant detected with Western blotting. **f** TEM observation of membrane vesicles prepared from ICD cells. The red box showed a representative vesicle. **g** The content of “eat me” signals in the membrane preparation of the ICD tumor cells detected with Western blot. Data are shown as the mean ± SEM. Statistical significance was calculated via one-way ANOVA (**d**), giving *P* values, **** *P* < 0.0001, *** *P* < 0.001, ** *P* < 0.01, * *P* < 0.05

To encapsulate PLGA particles with the membrane derived from the tumor cells undergoing ICD for the purpose to utilize the immunostimulatory characteristics of ICD cells and facilitate antitumor immune responses, at first the TC-1 tumor cells were treated with a combination of mitoxantrone (MTX) and curcumin (Cur). Observation of TC-1 cells treated with the drugs for 24 h showed a morphological change towards cell death under the microscope (Fig. 1a). The cells were stained with 7-ADD and Annexin-APC, and the results showed that the percentage of apoptosis-like cells increased gradually with the prolongation of the treatment time of MTX and Cur (Fig. 1b). The expression of “eat me” signals after the induction of ICD was observed by immunofluorescence. The results showed that the accumulation of CALR, HSP70 and HSP90 proteins was visualized at 6 h with a relative symmetrical distribution in the cell plasma, and the fluorescence signals along the cell edge became increasingly enhanced at 12 h and 24 h, which indicated that the “eat me” signals were expressed and localized at the surface of the cell membrane with the ICD induction (Fig. 1c). The levels of “find me” signals ATP and HMGB1 in the supernatant of treated tumor cells were measured dynamically at different time points. The results showed the release of ATP (Fig. 1d) and HMGB1 (Fig. 1e) in the supernatant was increased gradually with the induction of MTX and Cur from 6 to 30 h. Considering the “eat me” signals have been fully induced and the cellular state starts to get bad at 24 h, at this time point the tumor cells were collected for the membrane preparation. Transmission electron microscopy (TEM) observed that the preparation with the membrane extracts extruded through the extruder presented as nanoscale vesicles, the size ranging from 100 to 400 nm (Fig. 1f). Western blotting analysis showed the obviously higher contents of “eat me” signals CALR, HSP70 and HSP90 in the prepared ICD tumor cell membrane (IM) than those in the membrane vesicles of TC-1 cells without ICD induction, using Na^+^ K^+^/ATPase as a control (Fig. 1g). The above results indicated that the ICD of TC-1 tumor cells was induced with the release of “find me” signals and the membrane translocation of “eat me” signals, and the IM preparation was obtained successfully.

### Characterization of membrane-based biomimetic NPs

**Fig. 2:**
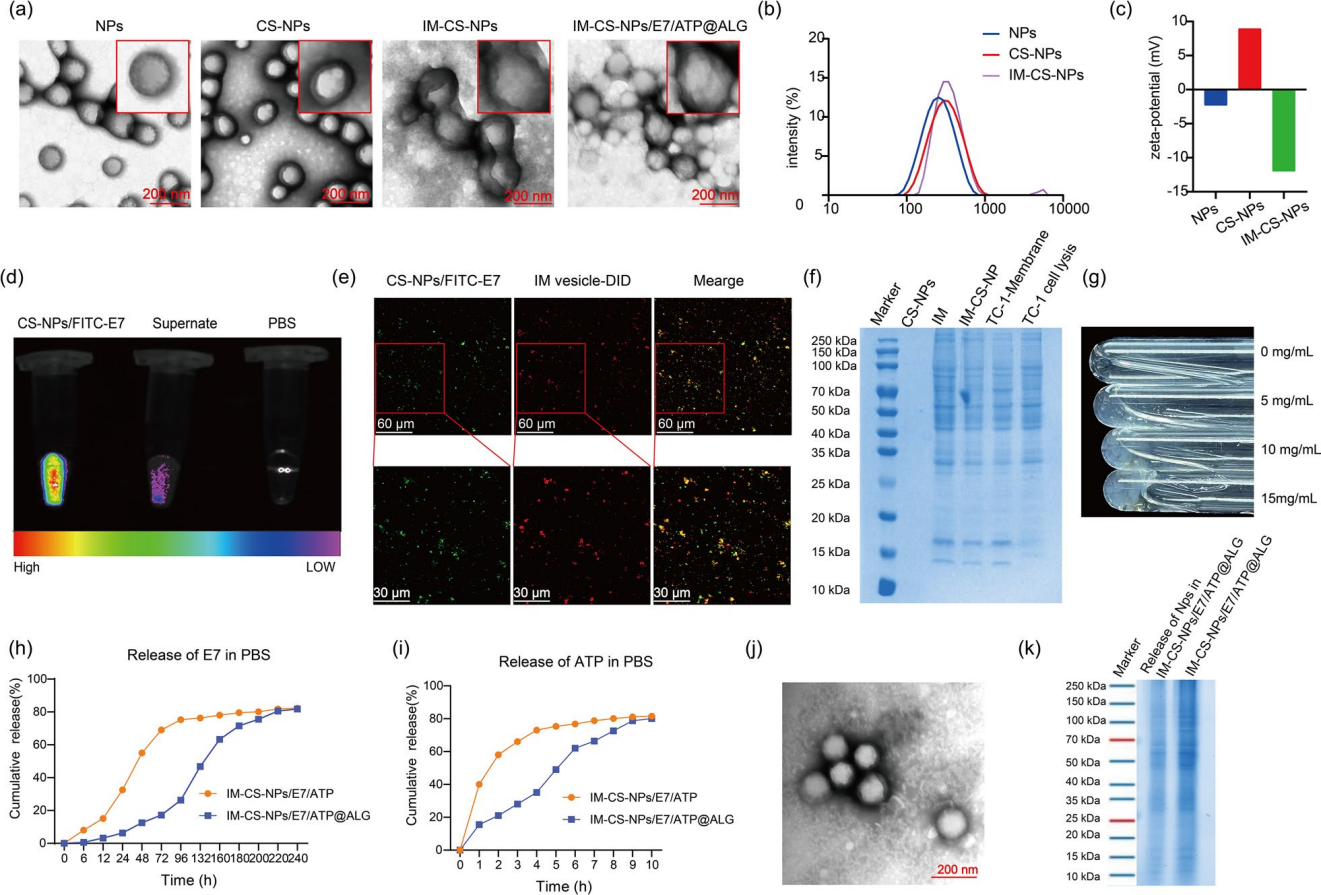
Characterization of membrane-based biomimetic nanovaccines. **a** TEM image of the NPs, scale bar = 200 nm. **b** Size distributions of the NPs detected by DLS. **c** Zeta potential analysis. **d** The encapsulation was demonstrated by detecting the fluorescence of CS-NPs/FITC-E7 and the supernatant of the second emulsion under the In-Fino FX PRO imaging system. **e** Confocal microscopy for analyzing the encapsulation of the NPs with ICD membrane. The E7 peptides and ICD membrane vesicles were labeled with FITC (green) and DID (red), respectively. **f** SDS-PAGE analysis on the distribution pattern of protein components. **g** Photograph showing the transition from ALG solution to hydrogel after the addition of CaCl_2_ solution. (h) E7 release profile from IM-CS-NPs/E7/ATP and IM-CS-NPs/E7/ATP@ALG in vitro. **i** Release curves for ATP in IM-CS-NPs/E7/ATP and IM-CS-NPs/E7/ATP@ALG in vitro. **j** TEM image of the NPs released from IM-CS-NPs/E7/ATP@ALG, scale bar = 200 nm. **k** SDS-PAGE analysis of the NPs released from IM-CS-NPs/E7/ATP@ALG

TEM and dynamic light scattering (DLS) were used to analyze the morphological characteristics and particle size distribution of the NPs. Under the TEM observation, PLGA NPs (NPs) showed a regular spherical morphology with a size ranging from about 100 to 150 nm, and chitosan PLGA nanoparticles (CS-NPs) had a similar morphology and size distribution to NPs but featured with a layer of translucent chitosan covered on the surface of PLGA NPs. IM wrapped chitosan PLGA nanoparticles (IM-CS-NPs) showed spherical shapes ranging from about 150 nm to 200 nm with a characteristic core–shell structure. PLGA nanoparticles in IM-CS-NPs/E7/ATP@ALG showed a regular spherical morphology with a size ranging from about 150 to 200 nm, which is similar to that of IM-CS-NPs (Fig. 2a). DLS analysis implied that the NPs were relative homogeneous with average sizes of 227.9 nm, 277.7 nm and 326.1 nm for NPs, CS-NPs and IM-CS-NPs, and PDI of 0.164, 0.184 and 0.165 for NPs, CS-NPs and IM-CS-NPs, respectively (Fig. 2b); and the zeta potentials on the surface of NPs, CS-NPs and IM-NPs were − 2.32 mv, 8.96 mv and – 12 mv, respectively (Fig. 2c). The analyses of particle morphology and zeta potential suggested that chitosan was efficiently loaded onto PLGA NPs and IM was also successfully wrapped on chitosan-covered PLGA NPs.

To further increase the antigenicity of the NPs, CS-NPs were loaded with tumor-specific CTL epitope HPV16 E7_44-62_ (CS-NPs/E7). FITC-E7_44-62_ peptides were used to replace E7_44-62_ for facilitating the detection of the encapsulation efficiency of peptides. The results of the very strong fluorescence intensity for CS-NPs/FITC-E7 and the extremely low intensity of free FITC-E7_44-62_ in the supernatant of the second emulsion (Fig. 2d) indicated that CS-NPs effectively encapsulated E7 peptides. And the encapsulation rate of E7 by CS-NPs was measured to be approximately 60% using an indirect quantitative method. To further demonstrate that ICD membrane was wrapped successfully onto CS-NPs, the CS-NPs-encapsulating FITC-E7 peptides were designated to indicate the NPs and DID red dye was used to labeled IM vesicles for confocal anlysis. After the co-extrusion of DID-IM and CS-NPs/FITC-E7 through the extruder, the yellow fluorescence produced by the overlap of green and red fluorescence was observed, which indicated that the CS-NPs were successfully modified with IM (Fig. 2e). Further, the distribution pattern of protein components in membrane vesicles and biomimetic NPs was analyzed by polyacrylamide gel electrophoresis (SDS-PAGE). Similar pattern of protein components to IM was found in IM-CS-NPs, supporting the conclusion that IM was coated on CS-NPs and also suggesting that the protein components on the tumor cell membrane keep stable during the wrapping process (Fig. 2f). To mimic the “find me” signals produced by ICD cells, the NPs were further equipped with ATP through embedding the NPs along with ATP into sodium alginate hydrogel, which may provide an effect of controlled release. The formation of hydrogels was observed in vitro by adding calcium chloride solutions, showing a certain degree of fluidity (Fig. 2g). Further, the release of E7 and ATP were analyzed using a simple dialysis experiment. The results showed that ATP was quickly released in IM-CS-NPs/E7/ATP in which ATP was just mixed with NPs, while E7 showed a clear controlled release as compared to ATP owing to the encapsulation of NPs; in addition, the formation of hydrogel in IM-CS-NPs/E7/ATP@ALG dramatically retarded the release of E7 and ATP, in comparison with IM-CS-NPs/E7/ATP (Fig. 2h, i). Further, the NPs released from the hydrogel of IM-CS-NPs/E7/ATP@ALG were collected by ultracentrifugation and observed with TEM and analyzed with SDS-PAGE. The results showed that the morphology (Fig. 2j) and the membrane protein constituents (Fig. 2k) of the NPs released from IM-CS-NPs/E7/ATP@ALG are similar to those of IM-CS-NPs/E7, implying the membrane on the surface of NPs remains intact while NPs are released from alginate hydrogel.

### Membrane-based biomimetic NPs promote the migration, uptake, activation and maturation of DCs cells in vitro

**Fig. 3:**
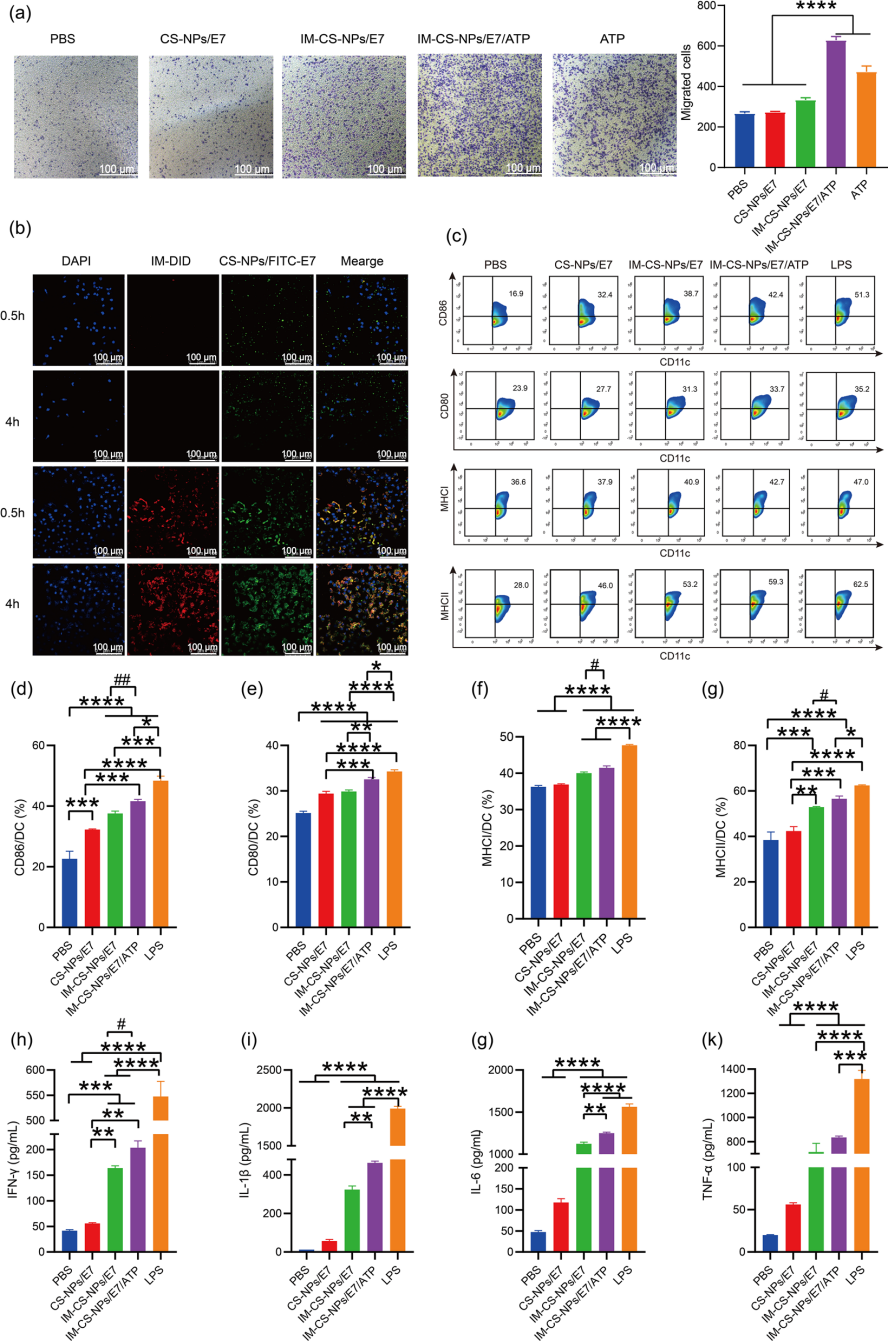
Membrane-encapsulated NPs stimulate migration, antigen uptake and maturation of BMDCs in vitro. **a** The Transwell cell migration assay (n = 3). **b** Uptake of the NPs by DC2.4 cells. The upper two panels are for the CS-NPs/FITC-E7, and the lower two panels are for the membrane-encapsulated nanoparticle DID-IM-CS-NPs/FITC-E7. **c** Representative plots of flow cytometry and (**d**–**g**) statistical analyses on the expression of maturation markers CD86 (**d**), CD80 (**e**), MHC I (**f**) and MHC II (**g**) on BMDCs (n = 5). **h**–**k** Proinflammatory Cytokine expression. IFN-γ (**h**), IL-1β (**i**), IL-6 (**j**) and TNF-α (**k**) levels in the culture supernatants were measured by ELISA (n = 3). Data are shown as the mean ± SEM. Statistical significance was calculated via one-way ANOVA, giving *P* values. **** *P* < 0.0001, *** *P* < 0.001, ** *P* < 0.01 (**d**–**k**); unpaired parametric t test, ^##^ *P* < 0.01, ^#^*P* < 0.05 (**d**, **f**–**h**)

To learn the potentials of membrane-based biomimetic NPs to serve as an effective vaccine, their effects on the migration and activation of DCs was evaluated in vitro. Migration of BMDCs cells was detected using a transwell migration assay. PBS, CS-NPs/E7, IM-CS-NPs/E7, IM-CS-NPs/E7/ATP and ATP were incubated with mouse bone marrow-derived dendritic cells (BMDCs) for 8 h. Representative images were taken at random under the light microscope (Fig. 3a). The results showed that IM-CS-NPs/E7/ATP had the strongest ability to promote the migration of BMDCs, while both free ATP and IM-CS-NPs/E7 also promoted BMDCs migration, and CS-NPs/E7 showed the similar result as PBS. The results demonstrated the chemotactic capacity of IM and ATP in the nanoparticle formulation, which was consistent with previous report [28, 31].

To track the cellular uptake of membrane-coated CS-NPs by DCs, The CS-NPs/FITC-E7 and DID-IM-CS-NPs/FITC-E7 NPs were incubated with DC2.4 cells respectively, and the uptake was analyzed with confocal microscopy. The results showed that a clear yellow fluorescence was visualized at 0.5 h and became more intense at 4 h (Fig. 3b), indicating that IM-CS-NPs/FITC-E7 exhibited quick and effective uptake by DC2.4 and also implying that IM did successfully encapsulate CS-NPs/FITC-E7 NPs. In a comparison, CS-NPs/FITC-E7 showed obvious but weaker than IM-CS-NPs/FITC-E7 green fluorescence. The results indicated that IM components and nanoparticle structure for IM-CS-NPs/FITC-E7 NPs produced an increased effect on promoting the antigen uptake by DC2.4 cells as compared to CS-NPs/FITC-E7.

Further, the effects of the NPs on DCs maturation and activation were evaluated through incubating with BMDCs in vitro for 24 h and analyzing the expression of co-stimulatory molecules on the cell surface by flow cytometry and the accumulation of pro-inflammatory cytokines in the culture supernatants by ELISA. (Fig. 3c) represents flow cytometry. The results showed that IM-CS-NPs/E7/ATP and IM-CS-NPs/E7 stimulated the maturation and activation of BMDCs more efficiently than CS-NPs/E7 as compared to the PBS control, evidenced by higher expression of CD86 (Fig. 3d), CD80 (Fig. 3e), MHCI (Fig. 3f), MHCII (Fig. 3g) and release of IFN-γ (Fig. 3h), IL-1β (Fig. 3i), IL-6 (Fig. 3j), TNF-α (Fig. 3k). By comparing between the two groups, IM-CS-NPs/E7/ATP showed a stronger effect than IM-CS-NPs/E7, indicating an adjuvant role of ATP.

### Membrane-based biomimetic NPs promote antigen lysosome escape in BMDCs and in vivo retention at injection site and accumulation in LNs

**Fig. 4:**
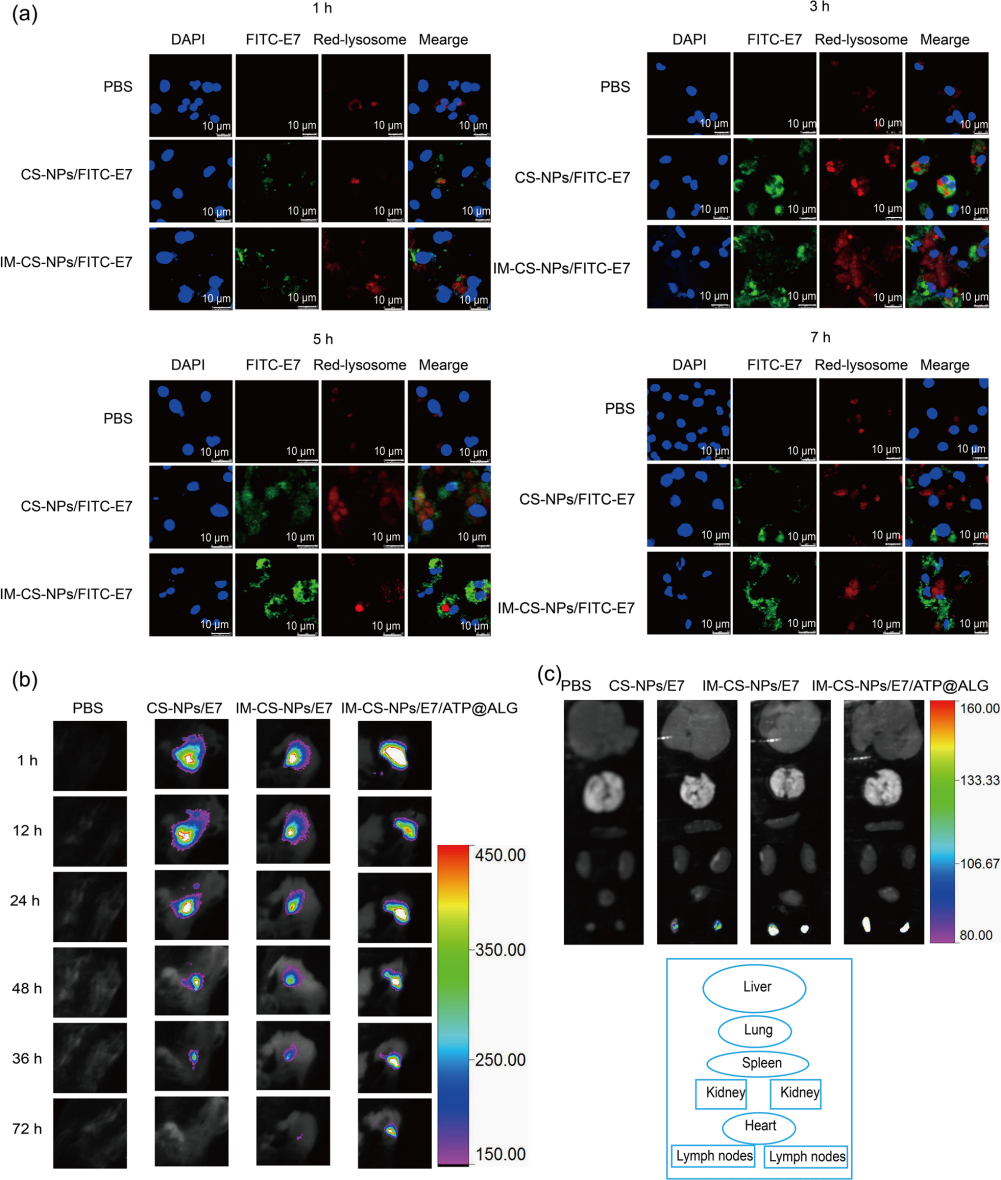
Membrane-encapsulated NPs promote lysosome escape in BMDCs and retention at injection site and accumulation in LNs. **a** Lysosome escape. E7 peptides were labelled with FITC and lysosome was visualized by staining with LysoTracker red dye. **b** Antigen residency at injection site detected by the In-Vivo FX PRO imaging system. **c** Ex vivo fluorescence images of main organs and inguinal lymph nodes for antigen biodistribution analysis

Cross-presentation is a key mechanism to promote antigen processing and presentation via MHC I molecule approach and generate cellular immune responses. The results of lysosome escape assay indicated both CS-NPs/FITC-E7 and IM-CS-NPs/FITC-E7 were efficiently taken in by BMDCs, which was consistent with the finding about IM-CS-NPs/FITC-E7 in DC2.4 (Fig. 3b). And IM-CS-NPs/FITC-E7 presented more intense signals than CS-NPs/FITC-E7, which indicated that the covered membrane components promoted the uptake efficiency of the CS-NPs/E7 NPs, which reinforced the related conclusion obtained from the experiment with DC2.4. In addition, it was found that there were obvious green signals existing in CS-NPs/FITC-E7 incubated cells in addition to yellow fluorescence, suggesting that except for a part of antigen dragged in endosome/lysosome system there were a large part escaped into plasma and might contribute to cross-presentation; noticeably, in IM-CS-NPs/FITC-E7 treated cells yellow fluorescence was seldom seen in the randomly selected fields of view, while the green fluorescence were intense, indicating that the encapsulating of membrane might further improved the capacity of NPs to escape from endosome/lysosome (Fig. 4a). The results together with the finding of enhanced expression of MHC-I strongly indicated the IM-encapsulated NPs was able to promote cross-presentation and might elicit a robust cellular immunity.

The persistent retention of antigen at the injection sites might provide an effect of controlled release and increase the period allowing the antigen to activate immune system. To examine the persistence of the vaccine formulation at the injection site and its biodistribution in vivo, CS-NPs/FITC-E7, IM-CS-NPs/FITC-E7 or IM-CS-NPs/FITC-E7/ATP@ALG was subcutaneously injected into the back of the mice, and the fluorescence intensity at the injection site was observed at designated time points using an In-Fino FX PRO imaging system (BRUKER, USA). The results showed that the fluorescence intensity at the injection site reduced as time went by in all the groups. However, the fluorescence signals of IM-CS-NPs/FITC-E7/ATP@ALG presented obvious more intense than the other groups 24 h after the injection; and the signals of CS-NPs/FITC-E7 was no longer detectable on day 3, and IM-CS-NPs/FITC-E7 remained a relatively weak signal at the injection site, while the IM-CS-NPs/FITC-E7/ATP@ALG still maintained a strong fluorescence signal (Fig. 4b). The results indicated that ALG hydrogel might provide a protective or controlled release effect on nanoparticle-encapsulated E7 antigen. In a parallel experiment, mice were sacrificed at 8 h and the heart, liver, spleen, lungs, kidneys and inguinal lymph nodes were collected, and fluorescence intensity was detected through ex vivo imaging using an In-Fino FX PRO imaging system. It was found that the fluorescent signal accumulated mainly in the lymph nodes in all the groups (Fig. 4c), but IM-CS-NPs/FITC-E7 and IM-CS-NPs/FITC-E7/ATP@ALG demonstrated a more distinctly intense fluorescence signal than CS-NPs/FITC-E7, which appeared to imply a role of membrane components covered on the NPs for DCs uptake or LN migration.

### The encapsulation of NPs with ICD cell membrane significantly enhanced the capability of the NPs to elicit antitumor immune effects

**Fig. 5:**
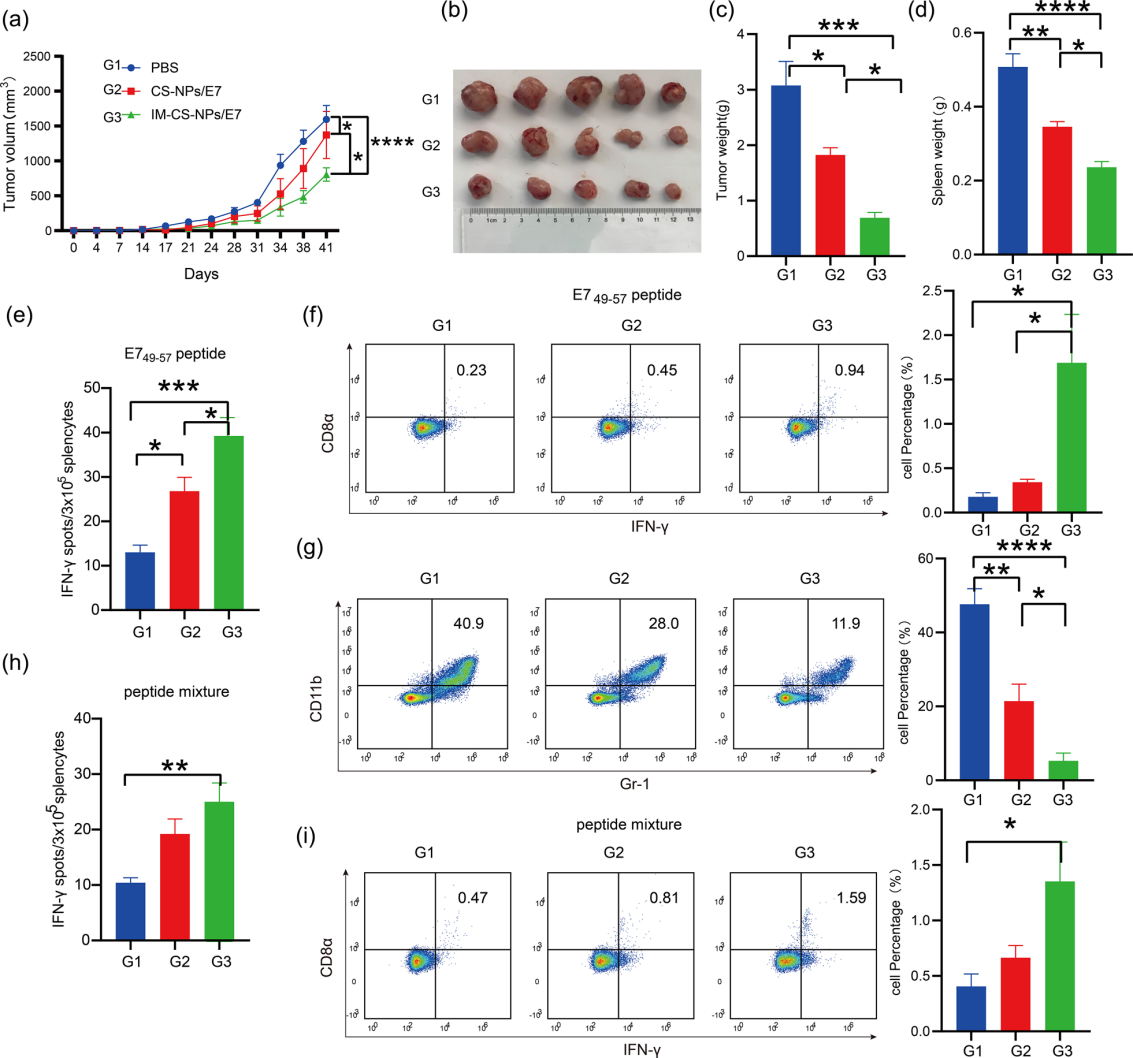
The encapsulation of NPs with ICD cell membrane significantly enhanced the capability of the nanoparticle to elicit antitumor immune effects. **a** Dynamic monitoring of tumor growth. **b** Representative picture of isolated tumor masses at the endpoint of the experiment. **c** Tumor weight. **d** Spleen weight. **e**, **h** Statistical analyses of ELISPOT, the isolated splenocytes were stimulate in vitro with E7_44-62_ peptide (**e**) or the mixture of E7 peptides (**h**). **f**–**g**, **i** Flow cytometry analyses of MDSCs (Gr-1^+^ CD11b^+^) (**g**), CD8^+^ splenocytes with a stimulation in vitro of E7_44-62_ peptide (**f**) and CD8^+^ splenocytes with a stimulation in vitro of the mixture peptides (**i**). Left panel, representative flow diagrams; right panel, statistical analyses. Data are shown as the mean ± SEM (n = 5). Statistical significance was calculated via two-way ANOVA (**a**) or one-way ANOVA (**c**–**i**), giving *P* values, **** *P* < 0.0001, *** *P* < 0.001, ** *P* < 0.01, and * *P* < 0.05

To evaluate the effect of modifying the CS-NPs/E7 NPs with ICD membrane, the mice were inoculated with TC-1 tumor cells first, and PBS, CS-NPs/E7 and IM-CS-NPs/E7 were administrated 3 times at an interval of 7 days after the tumor was fully established. Dynamic monitoring of tumor growth showed that IM-CS-NPs/E7 induced more significant tumor suppression than CS-NPs/E7 as compared to the PBS control (Fig. 5a). The result was well supported by the data on the size (Fig. 5b) and weight (Fig. 5c) of the tumor masses collected at the end of the experiment. Correspondingly, the splenomegaly induced by tumor growth was reduced in NPs-immunized mice (Fig. 5d).

Tumor antigen-specific IFN-γ-secreting lymphocytes represent an important population of antitumor effector cells. To clarify the possible cellular mechanism underlying antitumor effects of IM-CS-NPs/E7 immunizaion, the splenocytes were isolated and stimulated with specific antigenic peptides for ELISPOT and flow cytometry assays. In the experiments using E7_44-62_ peptide to stimulate splenocytes, the ELISPOT results showed that IM-CS-NPs/E7 stimulated a higher level of IFN-γ-secreting splenocytes than CS-NPs/E7 as compared to the PBS control (Fig. 5e); in addition, the frequency of CD8^+^ cells in spleen was significantly increased in IM-CS-NPs/E7 immunized mice in comparison with that in the mice receiving CS-NPs/E7 (Fig. 5f), while the frequency of immunosuppressive MDSCs (Gr-1^+^ CD11b^+^) was significantly reduced (Fig. 5g). To reflect the responses of broader antigen spectrum, a mixture of E7 peptides instead of E7_44-62_ was also used for the stimulation of the isolated splenocytes. Both ELISPOT for IFN-γ-secreting splenocytes (Fig. 5h) and flow cytometry for CD8^+^ cells in spleen (Fig. 5i) showed a similar response profile to that found in E7_44-62_-simulated cells. Overall, ICD tumor cell membrane-encapsulated NPs loaded with E7_44-62_ peptide were able to trigger a more vigorous anti-tumor immune response than the NPs without membrane encapsulation, indicating the importance of ICD membrane which provides “eat me” signals and possibly a broad spectrum of tumor antigens.

### Membrane-based biomimetic nanovaccine significantly inhibits the growth of established TC-1 tumor

**Fig. 6:**
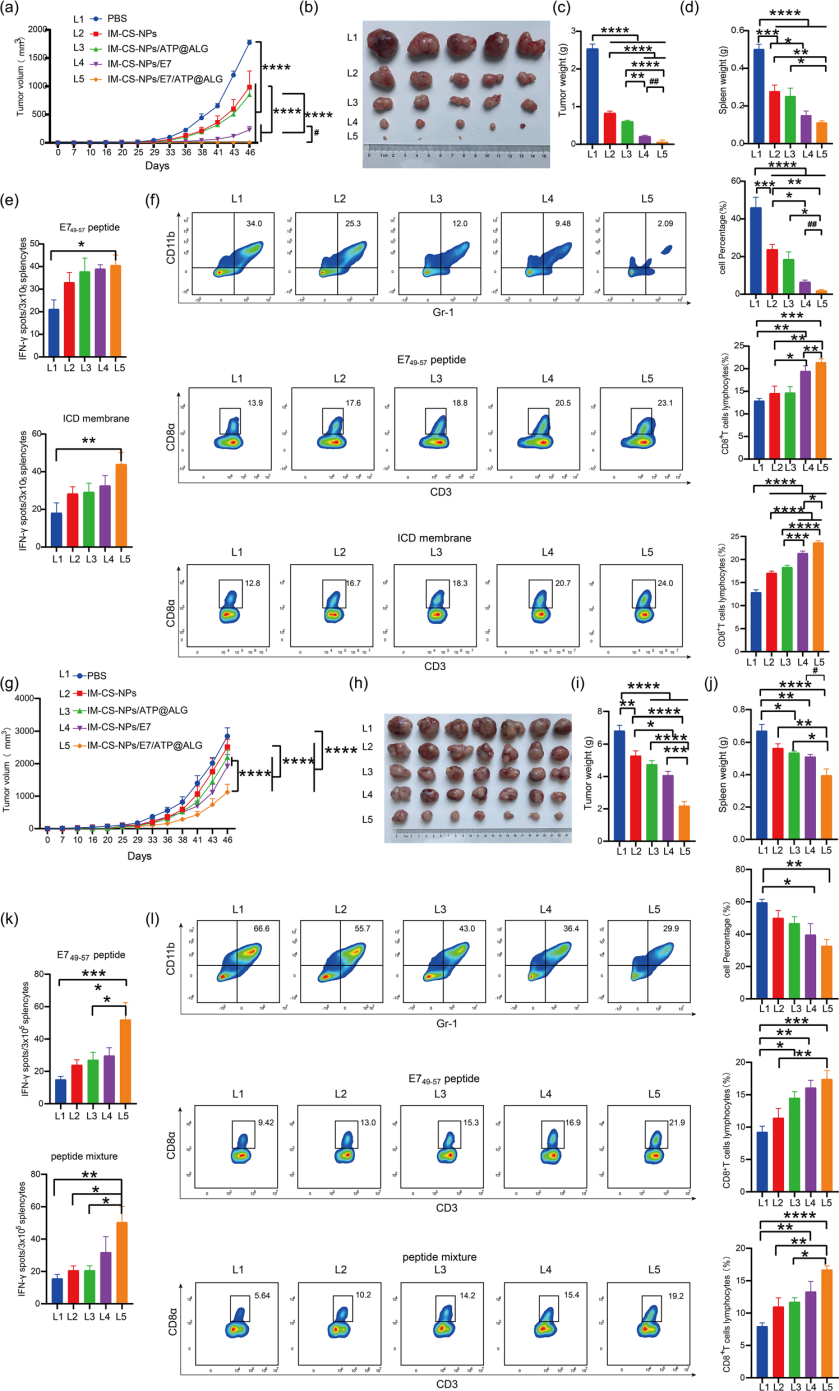
Membrane-based biomimetic nanovaccine significantly inhibits the growth of established TC-1 tumors. **a**, **g** Dynamic monitoring of tumor growth. **b**, **h** Representative picture of isolated tumor masses. **c**, **i** Tumor weight. **d**, **j** Spleen weight. **e**, **k** Statistical analyses of ELISPOT, the isolated splenocytes were stimulated in vitro by different stimuli. **f**, **l** Flow cytometry analyses for the levels of MDSCs (Gr-1^+^ CD11b^+^) and CD8^+^ cells. The isolated splenocytes were stimulated in vitro by different stimuli. Left, representative diagram; right panel, statistical analyses. In the two parallel experiments, the vaccination initialed when the tumor in mice grew to 2–3 mm, n = 5 (**a**-**f**) or 5–6 mm, n = 7 (**g**–**l**) respectively. Data are shown as the mean ± SEM. Statistical significance was calculated via two-way ANOVA (**a**, **g**) or one-way ANOVA (**c**–**f**, **I**–**l**), giving *P* values, **** *P* < 0.0001, *** *P* < 0.001, ** *P* < 0.01, and * *P* < 0.05. unpaired parametric t test, ^##^ *P* < 0.01, ^#^ *P* < 0.05 (**a**, **c**, **f**, **J**)

After the ICD membrane encapsulation for CS-NPs/E7 NPs was proven to significantly increase the capacity of the NPs to induce an anti-tumor immunity, a nanovaccine formulation (IM-CS-NPs/E7/ATP@ALG) fully simulating the immunostimulatory features of ICD cells was developed by further supplying a “find me” signal ATP with the embedding of ALG hydrogel. To assess the therapeutic potency of the nanovaccine and the importance of supplying ATP, the vaccination of IM-CS-NPs, IM-CS-NPs/ATP@ALG, IM-CS-NPs/E7 and IM-CS-NPs/E7/ATP@ALG NPs was perfomed after the tumor in mice grew to a size of 2–3 mm. Dynamic monitoring of tumor growth (Fig. 6a) showed that IM-CS-NPs/E7 more significantly inhibited the tumor growth than IM-CS-NPs and IM-CS-NPs/ATP@ALG, as compared to the PBS control; however, IM-CS-NPs/E7/ATP@ALG presented the most effective inhibition than all the other formualtions, even completely abolished the established tumor in two of five mice. At the end of the experiment on day 46, the tumor tissue and spleen of the mouse were separated. The results of tumour size (Fig. 6b), tumor weight (Fig. 6c) and spleen weight (Fig. 6d) were well consistent with the result of dynamic tumor growth curve. ELISPOT assay showed that the level of IFN-γ secreting splenic lymphocytes was significantly increased in the mice receiving IM-CS-NPs/E7/ATP@ALG vaccination, and the other fotmulations also a showed an increased tendency (Fig. 6e). Further, flow cytometry analyses demonstrated that the frequency of immunosuppressive MDSCs (Gr-l^+^ CD11b^+^) in splenocytes were more significantly lowered in the IM-CS-NPs/E7 and IM-CS-NPs/E7/ATP@ALG groups than IM-CS-NPs and IM-CS-NPs/ATP@ALG groups as compared to the PBS control, while IM-CS-NPs/E7/ATP@ALG group presented the most dramatical suppression; in contrast, both in specific peptide E7_44-62_ and tumor cell membrane components-stimulated splenocytes, the level of CD8^+^ cells was more significantly enhanced in mice receiving either IM-CS-NPs/E7 or IM-CS-NPs/E7/ATP@ALG than IM-CS-NPs or IM-CS-NPs/ATP@ALG, and still IM-CS-NPs/E7/ATP@ALG induced the most dramatical effects (Fig. 6f).

To further demonstrate the antitumor potency of the membrane-based mimetic nanovaccine IM-CS-NPs/E7/ATP@ALG, in a parallel experiment, larger tumors with a size of 5–6 mm was developed in mice before the immunization of the nanovaccine formulations. The results of tumor suppression and the responses of antitumor cellular immunity were similar to those found in the mice with 2–3 mm tumor (Fig. 6g–l); however, it was clearly indicated that larger tumor brought bigger challenge for the nanovaccines and IM-CS-NPs/E7/ATP@ALG induced more dramatical antitumor immunity and effects than the other nanovaccine formulations.

### Preliminary evaluation on the safety of the nanovacine

**Fig. 7:**
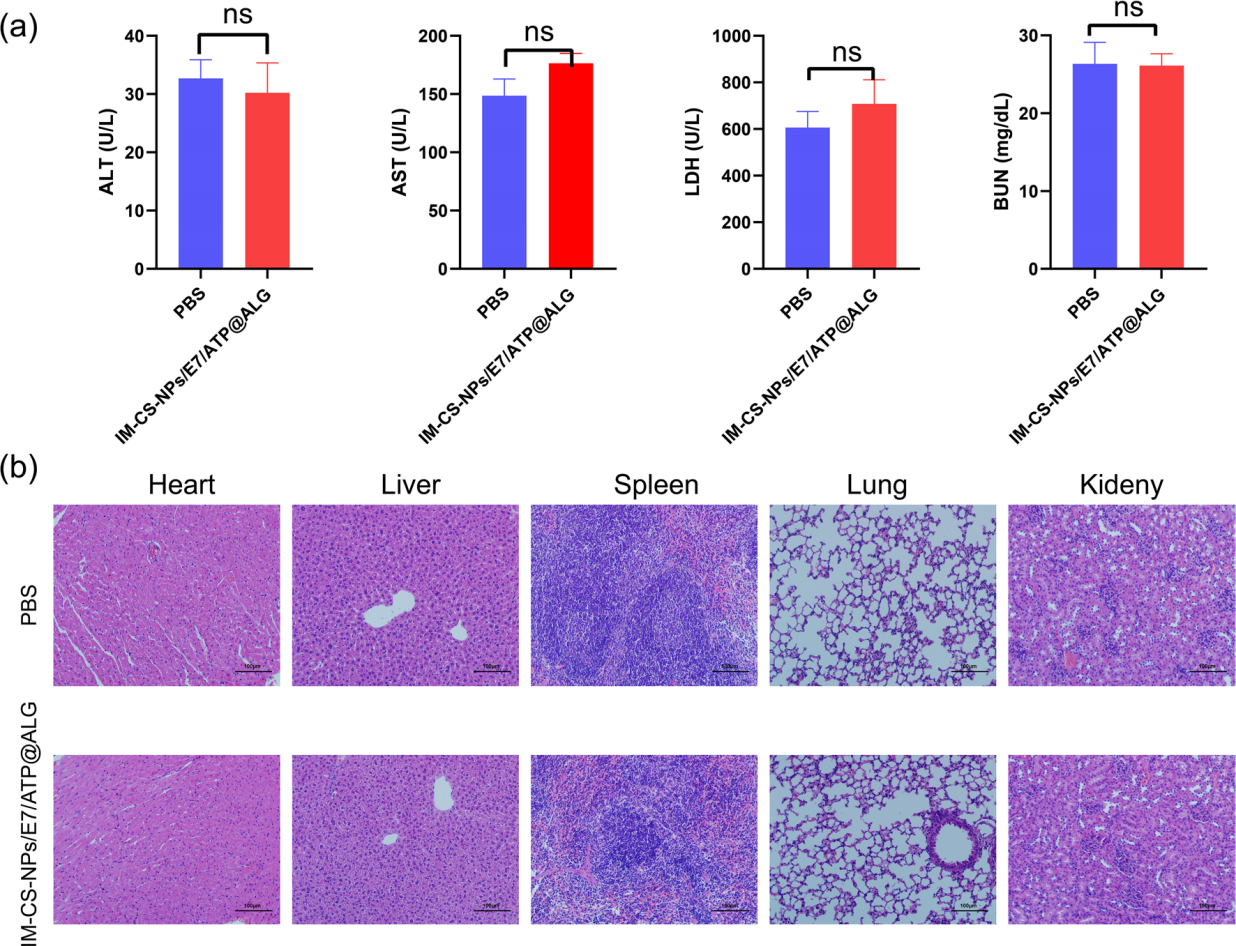
Preliminary safety evaluation of the nanovaccine IM-CS-NPs/E7/ATP@ALG. **A** Serum biochemical indices (n = 3). **B** Hematoxylin–eosin (H&E) staining of the tissue sections of major organs (n = 3). Data are shown as the mean ± standard error of the mean (SEM)

To Preliminarily evaluate the safety of the nanovaccine IM-CS-NPs/E7/ATP@ALG, the mice were injected with the vaccine formulation using the same protocol as that of the immunization experiment. The levels of the blood biochemical parameters including alanine aminotransferase (ALT), aspartate transaminase (AST), urea nitrogen (BUN) and lactate dehydrogenase (LDH) were not apparently changed in the mice injected with the nanovaccine, as compared to those receiving PBS; in addition, the tissue structure maintained normal and there was no inflammatory cell infiltration observed in H&E analysis. The results showed that the injection of IM-CS-NPs/E7/ATP@ALG didn’t cause apparent damage to major organs of the mice, preliminarily indicating a promising safety profile (Fig. 7).

## Discussion

Currently, the main clinical treatment means for cancers are still surgery, radiotherapy and chemotherapy, which may be not effective for recurrent or metastatic cancers as for early-stage cancers. Immunotherapy is an emerging strategy in cancer treatment, being prominent with recent landmark advances such as immune checkpoint blockade therapy and chimeric antigen receptor T-cell (CAR-T) therapy [32–36]. Tumor vaccine is a promising strategy for tumor immunotherapy, aiming to activate and enhance the normal anti-tumor immune responses of the immune system by effectively delivering specific tumor antigens and immune stimulators or regulators. The main challenges are the failure to develop sufficiently effective tumor-specific immune responses, the presence of a suppressive immune microenvironment and the antagonism of immune escape mechanisms [1, 37]. In particular, the genetic instability of tumor cells causes variation and heterogenicity of tumor antigens, which presents a main challenge for the screening and identification of specific antigens for vaccine application and limits the clinical efficacy of the current vaccine strategies [38]. Based on the fact that tumor cell membrane may possess a broad spectrum of tumor antigens, and the cooperation of multiple antigens is beneficial for the activation of different types and groups of APCs [39], the use of the tumor cell membrane provides an antigen strategy for developing a vaccine to cope with tumor heterogeneity.

In recent years, the membrane-based vaccine strategy has been developed rapidly. However, the low immunogenicity of the vaccine and the quality of the induced immune responses are still a main obstacle to gain clinical efficacy. One of the important concepts in the current study is to exploit the immunostimulatory mechanism of ICD tumor cells, which based on a finding that the outcome of clinical chemoradiotherapy is far more satisfactory if the ICD for tumor cells were induced. Further studies demonstrated that ICD tumor cells expressed a lot of DAMPs [40–42], some of which expose on the cell membrane, including CALR, HSP70 and HSP90, to act as “eat me” signals for APCs. Under steady-state conditions, CALR locates primarily in the lumen of the endoplasmic reticulum, acting as a molecular chaperone and regulating calcium signaling and homeostasis. the translocation of CALR to the surface of the plasma membrane and interacts with CD91 molecules on DCs cells, provide a mechanism to facilitate the uptake of cellular debris by APCs [43, 44]. Similarly, HSP70 and HSP90 translocate to the plasma membrane during tumor cell death and interact with receptors on the surface of the APCs [45], and also play a major role in the cross-presentation of tumor-derived antigenic peptides to CD8^+^ T cells, resulting in a cytotoxic response [46]. Another type of DAMPs during ICD is the released HMGB1 and “find me” signals ATP. ATP activates purinergic P2RX7 receptors on DC, thus activating the NLRP3/ASC/caspase-1 inflammasome and driving the secretion of interleukin-1β (IL-1β), which is required for the adequate polarization of IFN-γ-producing CD8^+^T cells [43]. Ultimately leading to CD8^+^ T cell activation and IFN-γ production [29, 47].

Therefore, in this study we employed ICD membrane of the tumor cells to provide the “eat me” signals and possibly a spectrum of tumor antigens, which enhanced uptake efficiency and activation of APCs; and, the encapsulation of PLGA NPs with the ICD membrane endowed the membrane components a more regular nanoscale framework, increasing the homogeneity of the membrane-based vaccine and further promoting the APC uptake and lymph node location. In addition, in this study, sodium alginate hydrogel was used to construct the nanovaccine formulation. The main function of hydrogels is to provide a controlled release of the enclosed contents such as antigen and adjuvant components. To fully simulate the immunostimulatory mechanism of ICD tumor cells, a “find me” signal ATP was supplied in the hydrogelated nanoparticle vaccine formulation, which promotes the recruitment and activation of nearby immune cells. We have showed that the formation of hydrogel in IM-CS-NPs/E7/ATP@ALG dramatically retarded the release of E7 antigen and ATP in vitro, in comparison with IM-CS-NPs/E7/ATP (Fig. 2h, i), and significantly prolonged the residence of antigen at the injection site in vivo and promoted the antigen accumulation in lymph nodes (Fig. 4b, c), which may facilitate the antigen uptake by antigen presenting cells and subsequent activation of adaptive immune cells. In a TC-1 tumor model with therapeutic intervention, IM-CS-NPs/E7/ATP@ALG elicited the strongest anti-tumor immune responses and produced the most significant suppression on the tumor growth among all the tested vaccine formulations. Taken together, our data indicated that the advantages of nanovaccines containing sodium alginate hydrogel over those without.

Another important concept highlighted in this current study is the codelivery of a representative tumor specific antigen with the tumor cell membrane using a nano-system. We expected that the immunodominant antigen peptide, together with the nanocarrier and ATP adjuvant, would efficiently elicit a specific immune response, which was supposed to also activate and modify the status and features of immune system besides providing the antigen peptide -specific anti-tumor effects; and the “warmed up” immune system would facilitate the antigen spreading i.e. the responses to the other epitopes for example those provided by the ICD membrane in our nanovaccine formulation. We provided some evidences to support this concept, for example, the membrane-encapsulated NPs still induced anti-tumor immune responses and significantly suppressed tumor growth even if the specific tumor antigen E7_44-62_ was not carried by the NPs (Fig. 6), implying the antigenicity of TC-1 tumor cells; and the inclusion of E7_44-62_ further enhanced the induced antitumor immunity with a broad antigen spectrum, which was evidenced by the increased responses to not only E7_44-62_ and also ICD membrane components.

Immunotherapeutic strategies based on tumor cell membrane NPs have proposed a new approach to develop personalized tumor vaccines. Large amount of cancer cell sequencing data show significant genetic heterogeneity among patients with the same type of cancer and even different cells in one individual. The personalized vaccines have the potentials to address the challenge raised by tumor heterogeneity [48–50]. This study further improved the clinical potency of the tumor cell membrane-based personalized nanovaccine, through simulating the immune characteristics of ICD tumor cells and describing a platform co-delivering membrane-associated antigens and the highlighted specific antigen or neoantigen.

Considering the immunosuppressive microenvironment of tumor, the efficacy of the membrane-based tumor vaccines may be further enhanced by a strategy in combination with the other treatment approaches. For example, a combination of immunization with tumor cell membrane-encapsulated NPs and checkpoint antagonism immunotherapy was shown to be more effective in improving anti-tumor responses, lowering tumor progressing and even inducing tumor elimination [16, 51]; in addition, the combination of vaccination with chemoradiotherapy, oncolytic virus therapy, photothermal therapy, or anti-cytokine active immunization has been proven to be synergically effective in preclinical studies. The underlying mechanisms mainly focus on the local intervening in tumor tissues to switch tumor microenvironment from immunosuppressive to immunoreactive profile, which facilitates to activating APCs, increasing antigen release, enhancing cross-presentation, and promoting the proliferation and activation, migration into tumor, and tumor-killing activity maintaining and cell survival of antitumor effector cells, and the effects well synergizes the main duty of a vaccine i.e. eliciting the generation of antigen-specific effector cells (Additional file 1).

In addition to the capacities for vaccine applications, membrane-based NPs can also be used as a platform for tumor-targeted delivery of drugs and immune modulators. Tumor cell membranes have the same cell adhesion molecules and receptors patterns as their source cells and thus have the similar specific targeting properties with highly self-selective targeting of homologous tumors in vivo, which may be used for membrane-decorated NPs to fulfill tumor-targeted intervening [52–54]. Efforts have been made to utilize the engineered membrane vesicles or membrane-decorated NPs to target and modulate the key immune cells such as macrophages in tumor microenvironment. For example, a programmable hybrid cell membrane vesicle using platelet-derived, macrophage-derived, and cancer cell-derived cellular nanovesicles enhanced the immune response of macrophages against cancer recurrence and metastasis [55]; a genetically programmable fusion cell vesicles (Fus-CVs) provided double blockade of CD47 and PD-L1 and significantly increased phagocytosis of cancer cells by macrophages, promoted antigen presentation, and activated anti-tumor T-cell immunity [56]; and, gene-edited cell membrane-coated magnetic NPs gCM-MNs effectively accumulated in the tumor microenvironment, blocked the CD47-SIRPα “don’t eat me” pathway, and repolarize tumor-associated macrophages (TAMs) to M1 phenotype, promoting macrophage phagocytosis of cancer cells and enhancing anti-tumor T cell immunity [57].

With a slight change for the nanoparticle formulation developed in this study, with ATP or/and other immune stimulators such as CpG, poly I:C, or MPLA, etc. encapsulated into PLGA NPs along with tumor-specific antigen, we may attain a local activation in tumor tissue, since our design targets simultaneously to both tumor immunosuppression mechanisms and heterogeneity and low immunogenicity of tumor antigen and thus holds the potency of overcoming tumor immunosuppression and inducing a broad spectrum of local and systemic antitumor immunity.

## Conclusions

In a summary, this study successfully developed a membrane-based biomimetic nanovaccine, which elicited a robust antitumor immune response and significantly suppressed tumor growth in the context of fully established tumor in mice. The nanovaccine utilizes the unique immunostimulatory mechanisms derived of immunogenic cell death, and fulfills a simultaneous nanoscale delivery of an highlighted tumor-specific antigen and broad membrane-associated tumor antigens, which presents the capacities to overcome tumor immune suppression and evasion, proposing an attractive strategy and delivery system for developing an effective tumor vaccine especially for personalized vaccine.

### Supplementary Information

**Additional file 1: **The gating method for flow cytometry.

## Data Availability

The data used and/or analyzed to support the current study are available from the corresponding author on reasonable request.
