# Supplementary material for: Exploiting immunostimulatory mechanisms of immunogenic cell death to develop membrane-encapsulated nanoparticles as a potent tumor vaccine
Source: J Nanobiotechnology. 2023 Sep 8;21:326. doi: 10.1186/s12951-023-02031-w (PMC10492316; doi:10.1186/s12951-023-02031-w)
Supplement: Supplementary file 1 — Additional file 1: The gating method for flow cytometry. [file 12951_2023_2031_MOESM1_ESM.docx]

The gating methods for flow cytometry analyses in figures 5 and figure 6 have been provided in supplementary data, as shown below.

a)


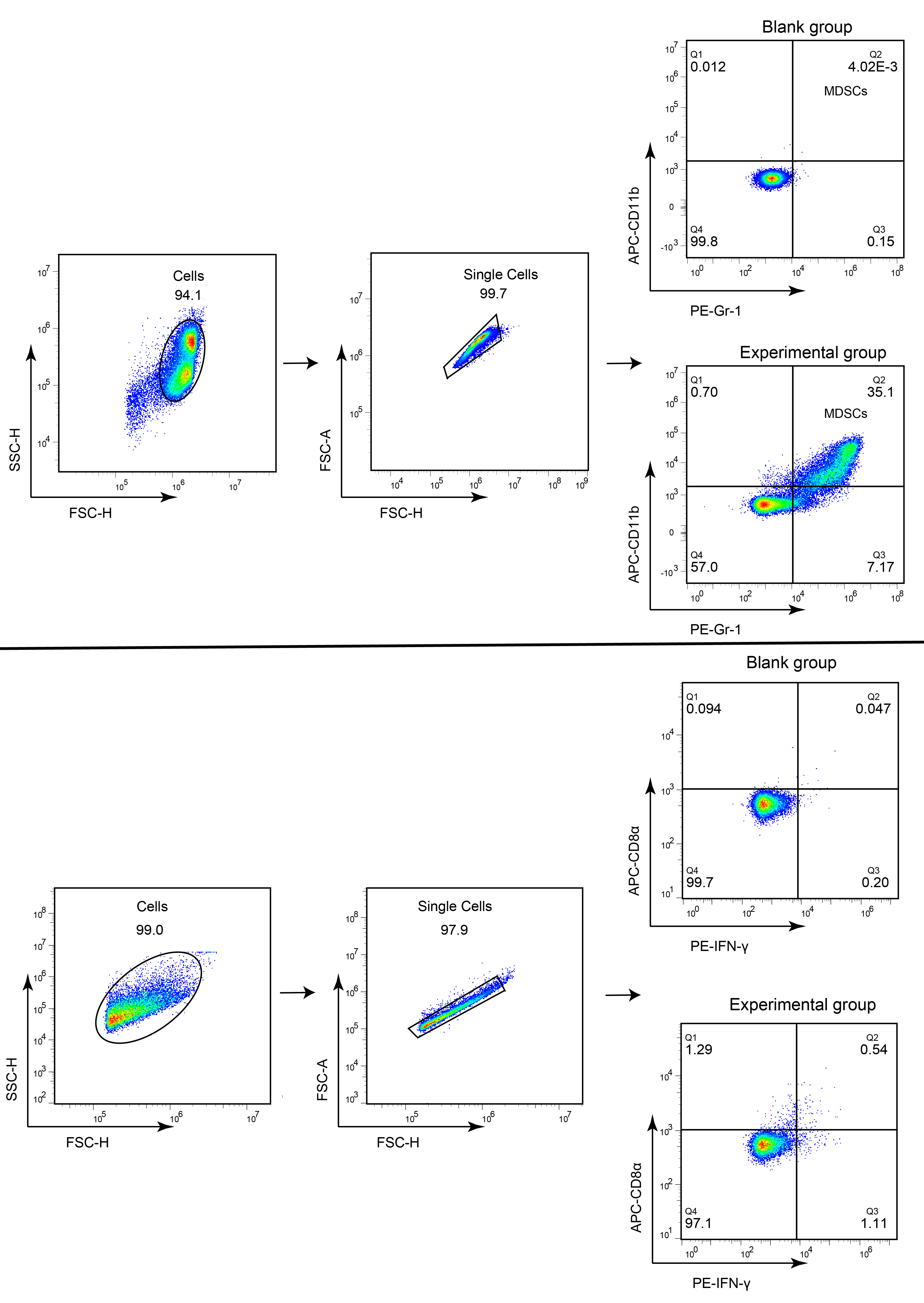


b)


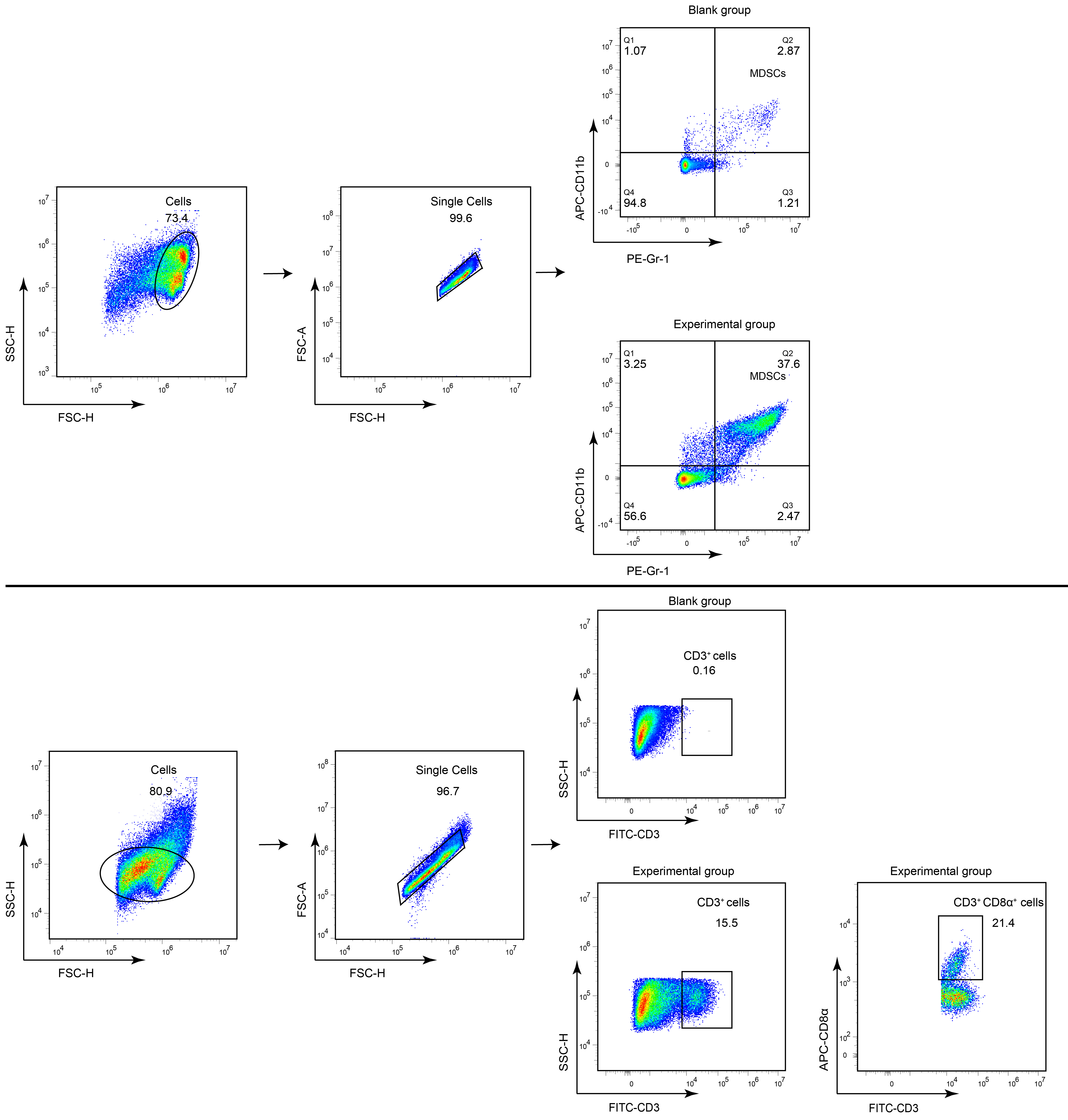


Supplementary data. The gating method for flow cytometry. a) the gating for figure 5; b) the gating for figure 6
